# Rhodiosin and herbacetin in *Rhodiola rosea* preparations: additional markers for quality control?

**DOI:** 10.1080/13880209.2019.1577460

**Published:** 2019-07-29

**Authors:** Zoltán Péter Zomborszki, Norbert Kúsz, Dezső Csupor, Wieland Peschel

**Affiliations:** a Faculty of Pharmacy, Department of Pharmacognosy, University of Szeged, Szeged, Hungary;; b Interdisciplinary Centre for Natural Products, University of Szeged, Szeged, Hungary;; c European Medicines Agency, London, UK

**Keywords:** HPLC, flavonoid

## Abstract

**Context:** *Rhodiola rosea* L. (Crassulaceae) is well-known to contain flavonoids such as the herbacetin derivative rhodiosin. However, flavonoids are not typically used in quality control.

**Objective:** This study analyses two flavonoids of *R. rosea* rhizomes and roots for their potential as analytical markers.

**Materials and methods:** Two constituents were isolated from ethanolic extracts via HPLC, identified via NMR and quantified via RP-HPLC. Presence and content variation was investigated according to extraction (solvent and repetitions), drying (temperature and duration) and sample origin (homogenously cultivated plants of different provenance, commercial samples).

**Results:** Rhodiosin was identified as a main flavonoid, accompanied by 10-fold lower concentrated herbacetin. Both compounds were best extracted with 70–90% ethanol, but were also detectable in more aqueous extracts. Different drying conditions had no effect on the flavonoid content. These two flavonoids were consistently identified in rhizome and root extracts of over 100 *R. rosea* samples. Rhizomes tend to contain less flavonoids, with average ratios of rosavins to flavonoids of 1.4 (rhizomes) and 0.4 (roots). Provenance differences were detected in the range (rhodiosin plus herbacetin) of 760–6300 µg/mL extract corresponding to a maximum of approximately 0.5–4.2% (w/w) in the dry drug.

**Conclusions:** For the first time, two main flavonoids present in *R. rosea* were quantified systematically. Rhodiosin and herbacetin can be detected simultaneously to phenylpropenoids or salidroside in authentic samples, influenced by the plant part examined and the plant origin. Rhodiosin and herbacetin may serve as additional marker to guarantee a consistent content of *R. rosea* products.

## Introduction

*Rhodiola rosea* L. (Crassulaceae) is one of the well-known plants of a group of so-called adaptogens. Derived products are used for various medicinal purposes linked to stress-related acute and chronic conditions, including mental and physical performance, ageing and cancer development (Chiang et al. 2015). Pharmacopoeia-specified standardization of products currently focuses on salidroside, a phenylethanoid found in all species of the *Rhodiola* genus besides some other plant species, as well as on phenylpropenoids characteristic of *R. rosea* and typically expressed as total rosavins.

Other constituents of *Rhodiola* species have occasionally been suggested to potentially contribute to pharmacological activities including the aglycon of the phenylpropenoids cinnamyl alcohol (CA) (Peschel et al. 2016), monoterpene glycosides such as rosiridin (van Diermen et al. 2009), gallic acid derivatives such as epigallocatechin-3-gallate (Chen et al. 2015) or lignans and some flavonoids including rhodiosin (**1**) (Zhou et al. 2015).

Flavonoids are common constituents of many herbal drugs, present mainly in traditional extracts using ethanol/water mixtures. Their reasonable stability and easy detection often make them the parameter of choice for standardization. Several flavonoids have been isolated from *Rhodiola* species and have been reported previously. These include herbacetin and its glycosides including 3,7-dimethylherbacetin, rhodiosin (herbacetin-7-*O-*glucorhamnoside), rhodionin (herbacetin-7-*O*-α-l-rhamnopyranoside), rhodalgin (herbacetin-8-*O-*α-l-arabinopyranoside), rhodionidin (herbacetin-*7-O-*α-l-rhamnopyranosyl-8-*O-*β-d-glucopyranoside), rhodalin (herbacetin-8-*O-*β-d-xylopyranoside), rhodalidin (herbacetin-3-*O-*β-d-glucopyranosyl-8-*O-*β-d-xylopyranoside), rhodiolin (a flavolignan of herbacetin); tricin and its glycosides, as well as the gossypetin glycosides rhodiolgin (gossypetin-7-*O*-l-rhamnopyranoside) and rhodiolgidin (gossypetin-7-*O-*α-l-mannopyranosil-8-*O-*β-d-glucopyranoside) (Kurkin et al. 1982, 1984a, 1984b; Zapesochnaya and Kurkin 1983; Zapesochnaya et al. 1985).

Regarding *R. rosea*, the isolated flavonoids are usually glycosides of kaempferol, gossypetin and herbacetin (**2**). In total, approximately 20 flavonoids have been described from this species, including tricin, herbacetin, gossypetin and their glycosides found in leaves/flowers/aerial parts, as well as flavonolignans and herbacetin found in underground parts, i.e., in rhizome or root (Zapesochnaya and Kurkin 1983; Zapesochnaya et al. 1985).

While some flavonoids, e.g., herbacetin are present in several families (e.g., Asteraceae, Gnaphilieae, Linaceae, Lauraceae, Atripliceae) (Wollenweber et al. 1997; El-sayed et al. 1999; Stewens et al. 1999; Fliniaux et al. 2014; Wei et al. 2017) others seem to be typical for *Rhodiola* species. These specific compounds, including rhodionin and rhodiosin, possess a specific sugar moiety such as the 3-*O*-β-d-glucopyranosyl-l-rhamnopyranose residue named ‘rhodiose’ after the first isolation works in Russia in the 1980s (Kurkin et al. 1982; Zapesochnaya and Kurkin 1983).

Like other flavonoids, herbacetin and derivatives show *in vitro* and *in vivo* effects that are linked to the multiple hydroxyl groups responsible for radical scavenging properties (Kwon et al. 2009; Qiao and Liu 2013) and associated with unspecific effects on membrane structures and enzyme activities related to cancer genesis and progression (Nakamura et al. 2007; Hyuga et al. 2013; Qiao et al. 2013), metabolism (Kobayashi et al. 2008) and other physiological and/or pathological processes (Jeong et al. 2009; Li et al. 2015, 2016).

While flavonoids in *R. rosea* are known, their quantity was never investigated in view of more unique and interesting characteristic constituents such as rosavin or salidroside. Hence, typical extracts for medicinal use have never been systematically studied in this regard either. In fact, flavonoids may not only contribute to some activities, but also give an additional analytical option to guarantee identity, purity and consistent content of medicinal products. Therefore, our research focused on identifying two main peaks with flavonoid UV spectra as potentially characteristic for *R. rosea* extract fingerprints following indicators from our previous studies. These two substances were isolated via HPLC and their structures were determined by NMR analysis and compared with literature data. We aimed to extend and validate a previously described HPLC method for the quantitative analysis in order to study the influence of extraction solvent, plant part, drying process and drug origin (different plant provenances, marketed drugs and products) on the flavonoid content. The overall goal of our study was to get a quantitative overview of the main flavonoids present in *R. rosea* in comparison to the conventional standards salidroside and rosavins.

## Materials and methods

### Plant and reference materials

Rhizome, root and herb samples were obtained from own cultivation as previously described (Peschel et al. 2016, 2018). Plants originate from diverse natural habitats, and voucher specimens have been deposited at the herbarium of the Biologiezentrum der Oberösterreichischen Landesmuseen (Linz, Austria).

Marketed commodities including four dried herbal drugs (I: ‘Rhizomata et radices Rhodiolae rosea’, 50 g cut pieces, Barnaul, Russia; II: unlabeled drug from a market stand, 40 g cut pieces, Barnaul, Russia; III: ‘Rhodiola rosea (rhizoma)’, 50 g cut pieces, Gorno-Altaisk, Russia; IV: ‘Rhodiolae radix concisus’, 500 g, Gittelde, Germany) and two products (I: Rhodiola rosea extract, 36.7 g/100 g powder in capsules, Germany); II: ‘Arctic root – Rhodiola rosea’, powder in capsules, originating from UK/sold in Szeged, Hungary) were studied. For the quantitative HPLC analysis, rhodiosin was purchased from Carbosynth (Compton, UK) and herbacetin was purchased from Phytolab (Vestenbergsgreuth, Germany).

### Processing and extraction

Plants were harvested, separated into different plant parts, cut, dried, stored, powdered and extracted as previously described (Peschel et al. 2013, 2016, 2018). Experimental variations in extraction/drying procedures or sample processing are explained below. The routine sample preparation for the quantitative analysis involves the following parameters: cutting before drying (2–6 cm, maximum 1 cm thick); drying temperature: 45 °C; drying duration: 6 days; powder particle size before extraction: 0.8–0.15 mm; extraction solvent: 70% ethanol (EtOH); drug to solvent ratio: 1:5; 5 days maceration at room temperature with shaking for 2 h at start and another 30 min at the end, followed by centrifugation.

### Isolation of flavonoids

For the isolation of flavonoids, 70% EtOH extracts of rhizome/root were combined. The isolation process was carried out using a Waters HPLC system comprising a Waters W600 pump, a W600 controller and a Waters 2487 dual channel UV detector, controlled by Empower software. A Kinetex XB-C18, (5 µm, 250 × 4.6 mm) column was used as the stationary phase. The mobile phase consisted of water (A) and acetonitrile (B). Elution was started with 83% A/17% B (1 min), then it was changed to 72% A/28% B (10 min) and to 50% A/50% B (5 min), followed by methanol washing for 5 min and another 5 min equilibration with the starting eluent. At 12 and 13 min, two main peaks were detected with UV spectra characteristic to flavonoids (UV *λ*_max_ 274, 328, 380 nm and 274, 328, 380 nm, respectively). These compounds were isolated by the HPLC method described above. Samples containing remarkable amounts of these compounds were pooled, evaporated and re-dissolved to gain a solution containing approx. 10 mg/mL of the index compounds. Forty injections (20 µL each) were carried out and the two peaks were separately collected. The purity of these two fractions was confirmed by HPLC and the identity of the evaporated compounds was elucidated by NMR.

### Identification of the isolated flavonoids

NMR spectra were recorded in methanol-*d*4 on a Bruker Avance 600 III spectrometer (^1^H: 600.13 MHz; ^13^C: 150.9 MHz) equipped with a 5 mm cryo-TXI probe. The peaks of the residual solvent (*δ*_H_ 3.31; *δ*_C_ 49.00) were taken as reference points. Chemical shifts are expressed in parts per million, and coupling constants (*J*) values are reported in Hz. Data were acquired and processed with the MestReNova v6.0.2-5475 software.

Compounds **1** and **2** were identified as rhodiosin and herbacetin, respectively, based on the comparison of their ^1^H and ^13^C spectral data with those in the literature (Nawwar et al. 1984; Jeong et al. 2009).

### HPLC quantitative analysis and validation

Rhodiosin (**1**) and herbacetin (**2**) were determined by external standard calibration using HPLC equipment, conditions and assay previously described, with an extension of running time and detection to 37 min (Peschel et al. 2016). Peaks were well separated and showed similar UV/VIS spectra (**1**: *t*_R_ 35.5 min, UV *λ*_max_ 274, 328, 380 nm; **2**: *t*_R_ 36.2 min; UV *λ*_max_ 274, 328, 380 nm). As the most suitable wavelength, UV *λ* = 254 nm was chosen for the analysis. Concentrations of **1** and **2** were determined in duplicate from three samples each, and expressed in μg/mL macerate (mean ± SD, *N* = 3). Experimental variations (if any) are given below.

#### Validation

Limits of detection (LOD) and quantification (LOQ) were evaluated by the Shimadzu^©^ LabSolutions (version 5.82) software. Precision was checked by repeated measurements of standards at a medium (100 μg/mL) concentration on the same day (intra-assay precision) and on three different days (inter-assay precision) (for R.S.D values see Table 1). Linearity was determined using six different concentrations per reference standard in the range of 6.16–308.0 μg/mL for **1** and 3.44–172.0 μg/mL for **2**, with a linear relationship as given in Table 1. Accuracy was checked by spiking of a flavonoid-low extract with 50%, 100% and 150% of the native amounts of **1** and **2**.

**Table 1.** Validation data for quantitative determination of rhodiosin (**1**) and herbacetin (**2**).

	*R* ^2^	Regression equation	Range (mg/mL)	LOD (μg/mL)	LOQ (μg/mL)	R.S.D.^a^ (%, *n* = 6)	R.S.D.^b^ (%, *n* = 6)
Rhodiosin	0.9989	*y* = 2E + 06*x* – 151221	0.00616–0.308	47.02	156.72	0.28	0.41
Herbacetin	0.9958	*y* = 2E + 06*x* + 336128	0.00344–0.172	7.60	25.35	1.38	2.47

^a^ Intra-assay precision within one analytical run 6 injections.

^b^ Between-assay precision at three different days (each sample in duplicate).

#### Derived parameters

Besides the absolute values of **1** and **2** (μg per mL liquid hydroethanol extract), we have also calculated the sum of both flavonoids (FLAV_tot_) and the ratio of flavonoids to phenylpropenoid, as well as the ratio of flavonoids to phenylethanoid compounds which are usually used for the standardization of *R. rosea*; these ratios served as relative parameters. Corresponding data for rosavins (expressed as ROS_tot_: sum of rosavin, rosarin and rosin) and their aglycon *trans*-CA (PP_tot_: total phenylpropenoids = total rosavins** **+** **CA), as well as for salidroside and its aglycon (SAL_tot_, sum of salidroside and tyrosol) were available and reported previously (Peschel et al. 2016, 2018).

For a comparison with literature data, we have also estimated the approximate amount of **1** and **2** in the original herbal drug (% w/w in the dry drug) based on the yield of the extraction process (see below) and on the drug to solvent ratio (% w/w flavonoids in dry drug = *x* mg/mL × 1.33 × 5 mL/g/10). For example, a tincture containing 1.0 mg/mL flavonoids approximately corresponds to a maximum of 0.66% flavonoids (w/w) in the original dry drug.

### Influence of solvent and extraction procedure

#### Influence of solvent polarity

Five different extracts were prepared (*N* = 3) using water, EtOH 30%, 50%, 70% and 90% v/v, respectively (all of analytical grade, Molar Chemicals, HU) for three drug samples of the same provenance (rhizome of a 4-year-old plant, UK cultivation; rhizome and root of a 6-year-old plant, Austrian cultivation) and flavonoid content was expressed in μg/mL (mean ± SD, *N* = 3).

#### Extraction process repetition

In order to check how efficient and exhaustive a single extraction process is, it was repeated thrice. The same three samples (two rhizome, one root) using four different extraction solvents (3 × 30% EtOH, 3 × 50% EtOH, 3 × 70% EtOH, 3 × 90% EtOH) were after the first extraction (M1) additional three times (M2–4) macerated with fresh solvent after filtering and drying the drug sample following the first and each following extraction. **1** and **2** contents (mean ± SD of measurement in duplicate of three samples each) of all four repetitions were compared.

### Influence of plant part used

From three authentic provenances (RR-I, RR-II, RR-III) and a previously identified non-authentic provenance (hybrid R-IV), three 5-year-old plants (three individual plants, each cultivated in the UK and harvested in July) were split into herb, rhizome and root, dried at 45 °C, ground (mesh diameter: 0.8–1.5 mm) and 5 g of each sample was finally extracted with 25.0 mL 70% EtOH for 5 days (see above). Contents of **1** and **2** were determined and expressed in μg/mL macerate (mean ± SD, *N* = 3).

### Influence of the drying procedure

*Drying temperature:* Two 5-year-old plants from five randomly chosen provenances (I–V, 7-year-old plants, cultivated in Austria, harvested in July) were split into rhizome and root. Each of the cut samples (2–6 cm, maximum 1 cm thick) were halved; one half was dried at 45 °C and the other one at 65 °C using warm air ventilation for 5 days, yielding four root and four rhizome samples at each temperature per provenance. Dry samples were ground and extracted as described above. Contents of **1** and **2** were determined and expressed in μg/mL macerate (mean ± SD of two plants, two samples each, *N* = 4).

*Drying duration:* Two 4-year-old plants from two randomly chosen provenances (VI–VII, 6-year-old plants, Austrian cultivation, harvested in October) were split into rhizome and root. Each sample was halved; one half was cut into smaller pieces (1–4 cm, maximum 0.5 cm thick) and the other half was cut into bigger pieces (3–8 cm, maximum 1.5 cm thick). All samples were split again, yielding four root and four rhizome samples for both sizes per provenance. Samples were dried at 20 °C at moderate air ventilation (fine-cut samples: 10 days, coarse-cut samples: 30 days). Dry samples were ground and extracted as described above. The content of **1** and **2** was determined and expressed in μg/mL macerate (means ± SD, *N* = 4).

### Influence of drug origin – commodities of commerce

Four marketed herbal drug samples (drug I–IV) and two products (prod I, II) were extracted as described above. In case of the commercial products, the powdered content of the capsules was directly used for extraction regardless of the excipients or any other possible ingredients contained. For each sample, two macerates were prepared and measured in duplicate.

### Influence of drug origin – plants of different provenances

Roots and rhizome samples from 9-year-old cultivated plants of 18 provenances (p01–p17 authenticated *R. rosea*, p18 an unknown species) and two wild alpine plants of unknown age (p19, p20) from the Eastern Alps were previously described and studied focusing on rosavins, CA and salidroside contents (Peschel et al. 2018). We grouped and analysed data as follows: (a) the average of the whole sample matrix of cultivated authentic *R. rosea* from 17 provenances, (b) means (*N* = 3,±S.E.M.) of all provenances (*N* = 20) and (c) means of five provenance groups: North Western European Islands (NW; *N* = 4), North Eastern Europe (NE, *N* = 3), Alps/Pyrenees (ALP/PYR; *N* = 6), Southern Siberia (ALTAI, *N* = 4), plus the two provenances from the Eastern Alps (wild Alp). The experimental design, sampling and analysis have been described previously (Peschel et al. 2018).

### Statistical analysis

*N* values for **1 **+** 2** were calculated as means of HPLC measurements carried out in duplicate. All samples tested were prepared in triplicate (with few exceptions prepared in duplicate only), and results were calculated as mean ± SD per treatment/variable factor. For provenance comparison, means ± S.E.M. were calculated for (I) all individual plants (*N* = 51), (II) three individual plants of each genotype (*N* = 17) and (III) five geographical region groups with divergent *N*. For geographical region groups, significance was tested for each compound parameter using two-way ANOVA with or without Tukey’s post-test (R-3.2.1 software) and results are indicated with different letters for those groups with *p* < 0.05.

## Results and discussion

### Isolation, identification and assays of 1 and 2

**Figure 1.**
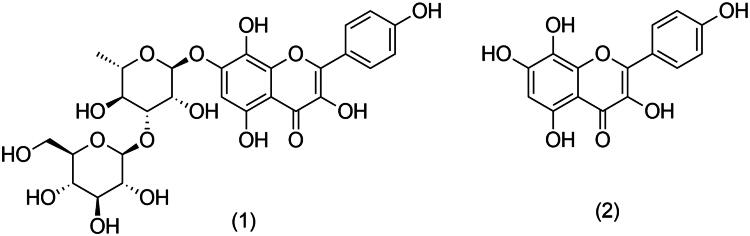
Structure of rhodiosin (**1**) and herbacetin (**2**).

The isolation procedure using a dry pooled extract (root plus rhizome) yielded 5.24 mg of **1** and 3.41 mg of **2**. Preliminary identification of the compounds was carried out by comparing the UV spectra of **1** and **2** with analytical standards. Structure validation was carried out via NMR analysis using a Bruker Avance 600 III spectrometer (^1^H: 600.13 MHz; ^13^C: 150.9 MHz). Although the presence of several other flavonoids has been reported for *Rhodiola* species previously, we focused on **1** and **2** for possible quantitative analysis (Figure 1).

**Figure 2.**
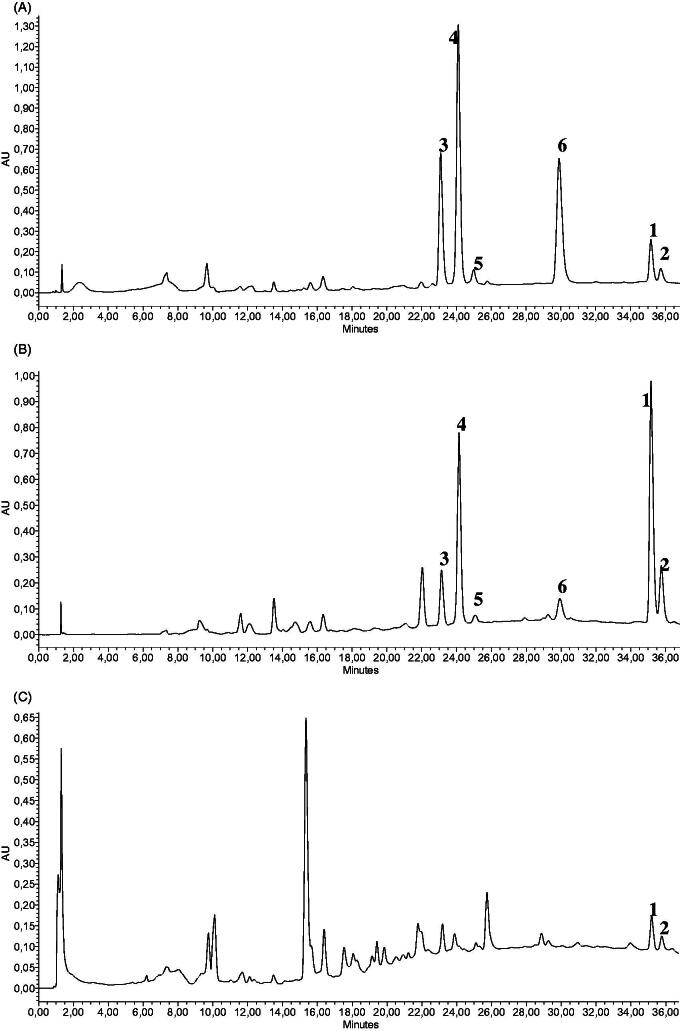
HPLC chromatogram (*λ* = 254 nm) for the simultaneous determination of 1 and 2 alongside characteristic phenylpropenoids in *R. rosea* (**1**, rhodiosin; **2**, herbacetin; **3**, rosarin; **4**, rosavin; **5**, rosin; **6**, cinnamylalcohol). (A) Authentic *R. rosea* rhizome; (B) authentic *R. rosea* root; (C) non-identified *Rhodiola* species root.

Via the extension of our previously described HPLC method (Peschel et al. 2016), **1** and **2** (*t*_R_: 35.5 36.2 min, respectively, Figure 2) could be detected baseline-separated from other peaks. Despite a prolonged run time of the analysis, simultaneous detection to rosavins and salidroside is advantageous for a HPLC fingerprint, as well as for quantitative determination.

Regarding the assay, detection may be accomplished at several wavelengths including 254 nm (used for rosavins and CA detection) and 275 nm (used for salidroside and tyrosol detection) without major differences in results despite baseline deviations. Precision, linearity and accuracy were checked for both 254 nm and 275 nm detections (Table 1). The LOD was 47.02 μg/mL and 7.60 μg/mL for **1** and **2**, respectively, with a signal to noise ratio of ≥3. The LOQ was 156.72 μg/mL and 25.35 μg/mL, respectively, with a signal to noise ratio of ≥10. Recovery rates were 84.66, 89.51 and 93.25% for **1** and 56.42, 64.99 and 75.54% for **2** at 50, 100 and 150%, respectively.

### Extraction of 1 and 2

**Figure 3.**
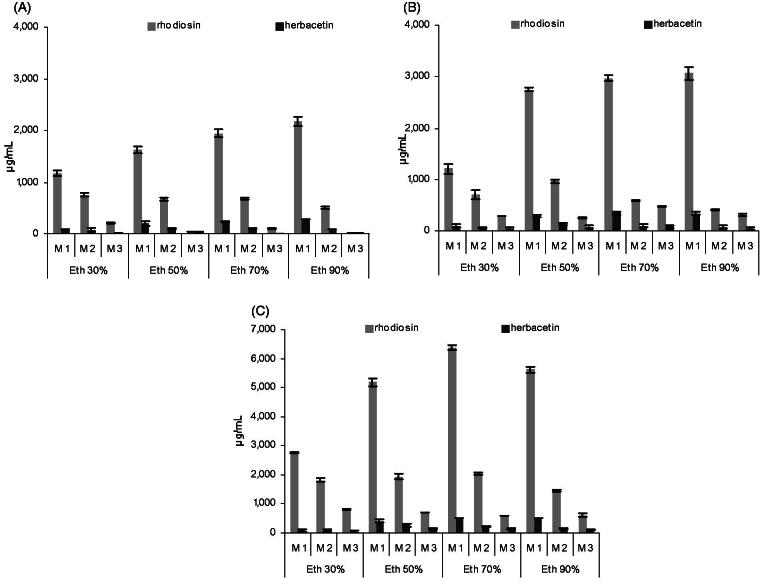
Influence of the extraction solvent on extraction efficiency. Contents of **1** and **2** in rhizome (A, B) and root samples (C), each extracted with four different solvents and three successive macerations M1–M3 (*n* = 3 each, mean ± SD).

Traditional *Rhodiola* tinctures and dry extracts used in contemporary products are commonly based on hydro-ethanol extraction. Regarding the tested range of five solvents, the flavonoids of interest were best extracted with 70–90% EtOH, slightly less with 50% EtOH and considerably less with 30% EtOH (Figure 3). Flavonoids were still detectable in pure aqueous extracts, but were below the LOQ in our setting (not included in graph). These findings suggest that commonly available extracts (40–70% EtOH) should contain detectable amounts of flavonoids besides rosavins and salidroside, but these may not be optimally extracted. We used 70% EtOH for our screening, which is generally used for an optimized phenylpropenoid yield as previously reported. We found a higher flavonoid content in roots than rhizomes independent of the extraction solvent, while phenylpropenoids were found to be less concentrated in the roots (Peschel et al. 2016).

Using 70–90% EtOH, approximately 75% of the total extractable amount was obtained in a single extraction process (5-day maceration). A nearly exhaustive extraction was reached with two follow-up macerations. A fourth repetition did not yield relevant extra amounts (<1% of total yield) and was often below the LOQ. These data indicate that for the maximum extraction of **1** and **2** from dried plant samples 70–90% EtOH and 2–3 repeated extractions may be appropriate, allowing to determine the total flavonoid content which could be suitable, e.g., for plant characterization or root/rhizome assays for pharmaceutical starting materials or powdered drugs directly used in commercial products. For our screening purpose, the single maceration, yielding consistently about 75% of the total flavonoid content was considered to be suitable to compare the 70% EtOH extracts of tested samples.

### Flavonoid content of different plant parts

**Figure 4.**
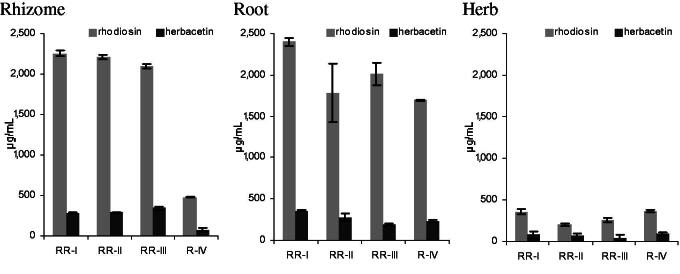
Flavonoid concentrations in 70% EtOH extracts from rhizome, root and herb of four different provenances (*N* = 3 each, mean ± SD).

The flavonoid content of the herb was found to be rather low (<400 μg/mL in the extract, corresponding to cca. 0.2% in the dry drug) compared to rhizome and root, whose extracts usually contained at least 1800–2400 μg/mL of flavonoids (corresponding to 1.2–1.6% in dry drug) for our sample set (Figure 4). Irrespective of the plant part analysed, both **1** and **2** are always detected, and **1** is usually 5- to 10-fold concentrated compared to **2**.

A hybrid accession (R-IV) previously described as having deviating morphological characteristics and exceptionally low amounts of phenylpropenoids (Peschel et al. 2013, 2016) also contained **1** and **2** in the rhizome in lower concentrations than in the authentic provenances. Consequently, testing simply for the presence/absence of **1** and **2** (as a qualitative fingerprint) may not be suitable for identification of the species; however, when quantified, particularly low amounts of flavonoids can indicate quality issues such as admixtures of other *Rhodiola* species. In contrast to other root/rhizome comparisons in this study, amounts of **1** and **2** were largely equal in both plant parts, while elsewhere root extracts exhibited often more than double the amount than rhizome extracts. Reasons for these differences are not obvious, but this phenomenon might be linked to a deviating cultivation site and harvest time.

### Influence of the drying procedure

**Figure 5.**
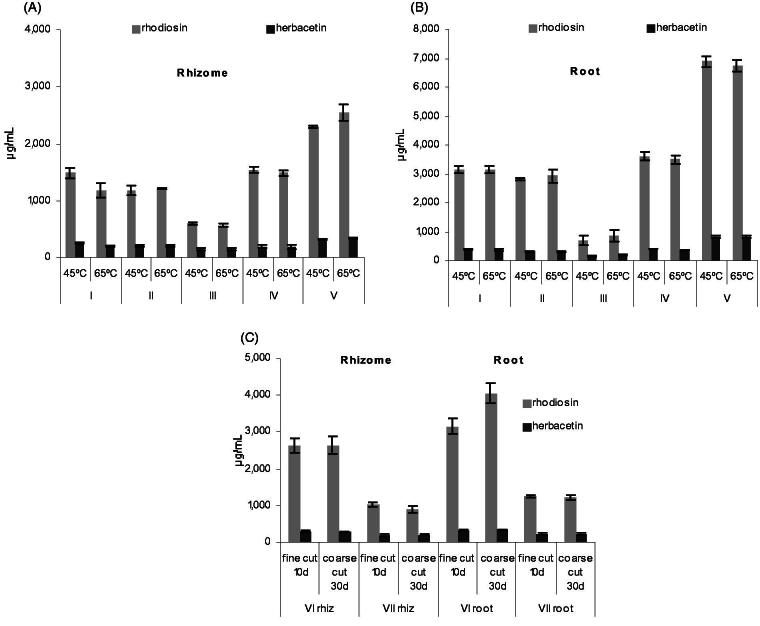
Flavonoid concentrations in rhizome and root extracts (70% EtOH) according to (A + B) drying temperature for five different provenances (I–V) and (C) duration of drying for two different provenances (VI–VII) (*N* = 3 each, mean ± SD).

Different temperatures applied during drying (45 versus 65 °C for 5 days), as well as different drying durations according to cut size (10 days versus 30 days at room temperature) did not affect the flavonoid content (Figure 5). Contents of **1** and **2** were more influenced by plant provenance and the plant part used. The largely consistent ratios of **1** and **2** also suggest that both flavonoids are stable and are not affected by drying temperature or potential post-harvest enzymatic degradation.

### Flavonoid content of marketed commodities

**Figure 6.**
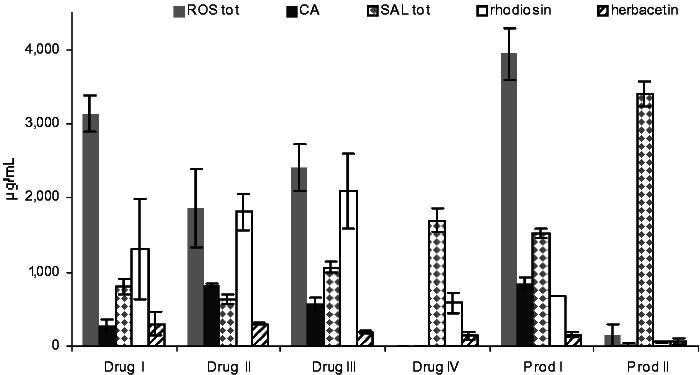
Concentrations of **1** and **2** in 70% EtOH extracts from marketed commodities (four dried herbal drugs and two products) of different origin, also showing their content in rosavins (ROS_tot_), cinnamyl alcohol (CA) and salidroside (SAL_tot_) (*N* = 2, mean ± SD).

In all samples of marketed *Rhodiola* drugs and products **1** and **2** were detected in very different quantities (Figure 6). For a better comparison of extract profiles, the flavonoid content is presented alongside previously determined contents of rosavins, CA and salidroside. Three drug samples originating from Russian markets and shops containing a visibly major rhizome portion showed similar profiles. In contrast, the drug sold as Rhodiolae radix concisus had lower amounts of **1** and **2** (less concentrated than salidroside), but most obviously it contained no phenylpropenoids. This finding confirms that ‘Rhodiolae radix’ does not equal ‘Rhodiolae roseae rhizoma et radix’, and that other *Rhodiola* species are also marketed without specifying the exact species. Also product I (a mixture of powder and a 70% EtOH extract) was found to have a moderate flavonoid content, whereas product II showed very low amounts of **1**, **2** and phenylpropenoids, but a high salidroside content. In this case, it is assumed that *R. rosea* is only a minor part of the active substance, while either salidroside is added or other *Rhodiola* species prevail despite the product being labelled as *R. rosea*.

### Flavonoid content in homogenously cultivated plants of different provenances

On average, rhizome and root extracts of 51 simultaneously harvested 9-year-old plants (cultivated in the Eastern Alps) from 17, mainly European provenances contained 1800 and 3100 µg/mL flavonoids, respectively (Table 2). This means that our extraction method yielded rhizome extracts with slightly more species-characteristic rosavins than flavonoids, while root extracts contained more flavonoids than rosavins. Since phenylpropenoid values (ROS_tot_** **+** **CA) are usually higher in rhizomes than in root, as previously reported, the ratio of rosavins to flavonoids differ substantially between these two plant parts (1.4 and 0.4, respectively). Notably, in our 70% EtOH extracts the quantities of flavonoids usually surpass those of salidroside, the common standardization parameter for all *Rhodiola* species.

**Table 2.** Content of **1** and **2** in 70% EtOH extracts from rhizomes and roots of *R. rosea* cultivated at 1580 m in Eastern Austria (*n* = 3, mean ± S.E.M., range of 51 samples across provenances p01–p17 harvested in May year 9).

	Rhizome		Root	
	Mean (±S.E.M.)	Range (min–max)	Mean (±S.E.M.)	Range (min–max)
Rhodiosin (**1**) (μg/mL)	1586 (±211)	159–4885	2817 (±450)	216–8606
Herbacetin (**2**) (μg/mL)	211 (±20)	53–547	308 (±34)	69–862
FLAV_tot_ (**1 **+**2**) (μg/mL)	1797 (±230)	220–5431	3125 (±482)	285–9468
ROS_tot_^a^^^(μg/mL)	1842 (±207)	530–4273	980 (±118)	361–2494
PP_tot_^b^^^(μg/mL)	2189 (±239)	729–5460	1236 (±140)	390–2844
SAL_tot_^c^^^(μg/mL)	547 (±123)	112–3079	332 (±75)	54–1503
ROS_tot_/FLAV_tot_ ratio (*x*:1)	1.39 (±0.25)	0.30–4.84	0.44 (±0.09)	0.10–1.54
SAL_tot_/FLAV_tot_ ratio (*x*:1)	0.41 (±0.17)	0.06–4.91	0.21 (±0.14)	0.01–4.05

Comparison to previously detected phenylpropenoids and phenylethanoids plus derived ratios.

^a^ ROS_tot_ = rosavin + rosarin + rosin.

^b^ PP_tot_ = ROS_tot_ + aglycon cinnamyl alcohol (CA).

^c^ SAL_tot_ = salidroside + aglycon tyrosol.

**Figure 7.**
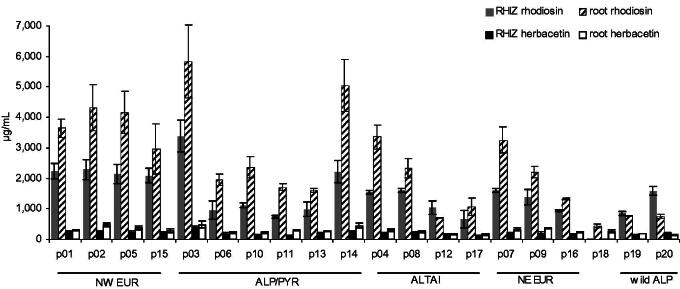
Concentrations of **1** and **2** in roots and rhizomes of 17 provenances of *R. rosea* cultivated at 1580 m in Eastern Austria (p01–p17, *N* = 3, mean ± S.E.M. harvested in year 9), as well as in p18 (unknown *Rhodiola* species) and p19/p20 (*R. rosea* of unknown age from wild collection) used as comparators. Provenances are grouped according to the main area of origin: North Western European Islands (NW), North Eastern Europe (NE), Alps/Pyrenees (ALP/PYR), Southern Siberia (ALTAI), direct samples from the Eastern Alps (wild Alp).

As per provenance, mean flavonoid values were in the range of 760–3800 µg/mL in rhizomes and 880–6300 µg/mL in roots (Figure 7). Thus, **1 **+** 2** (with a consistent ratio of about 10:1) were detected in all cultivated provenances and in those collected from the wild. They were also found, but in lower amounts, in the root extracts of the unidentified species p18, albeit with a divergent ratio of **1** and **2** (1.6:1). Provenances with a particularly high flavonoid content in rhizome (>2000 µg/mL) or root (>3000 µg/mL) or in both, originate from diverse locations such as the Pyrenees (p03), the Alps (p14), NW European Isles (p01, p02, p05) and Russia (p04, p07). Also those with a lower flavonoid content (≤1000 µg/mL in rhizome or ≤2000 µg/mL in root) are from different habitats: Southern Siberia (p12, p16), Finland (p16) and all cultivated and collected plants from the Alps (p06, p10, p11, p13, p19, p20).

**Figure 8.**
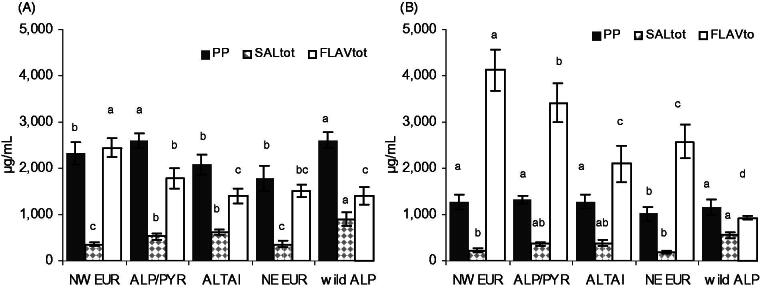
Flavonoids (FLAV_tot_ = **1** + **2**), rosavins plus aglycon CA (PP_tot_) and salidroside plus aglycon tyrosol (SAL_tot_) in rhizomes (A) and roots (B) of *R. rosea* (cultivated at 1580 m, harvested in year 9; 70% EtOH extracts, mean ± S.E.M.) according to the main area of origin. Significance (two-way ANOVA plus Tukey’s post test) is indicated with different letters when *p* < 0.05.

Despite such diversity found according to regional groups, the rhizomes and roots of *R. rosea* of NW European origin contained significantly more flavonoids than any of the other four groups (Figure 8). In Alpine provenances, it was generally low, but mean values were affected by two obvious high-flavonoid outliers from the Pyrenees and Swiss Alps. It suggests that provenance does play a role, i.e., plant genetics might influence the flavonoid content. However, the groups arbitrarily formed according to the main geographical area of origin may not be suitable alone to predict flavonoid contents. More provenances, ideally of the same age and cultivated under equal conditions, are required to confirm the trends observed here. Local variants with particularly high or low quantities of flavonoids can be assumed.

In order to understand the relevance of flavonoid quantification for analytical standardization, we compared the amounts of the two measured flavonoids with the levels of phenylpropenoids (rosavins plus aglycon CA) and salidroside (plus aglycon tyrosol) (Figure 8). For all regional groups the flavonoid content of rhizome extracts was in a similar range as that of rosavins (PP_tot_/FLAV_tot_ ratio 0.9–1.8). In root extracts, flavonoids had at least double amounts of rosavins across all cultivated geographical sources with the exception of the plants collected from the wild (PP_tot_/FLAV_tot_ ratio 0.3–1.2).

These results emphasize the relevance of rhizome/root portions in a given drug. Nonetheless, the ratios of flavonoids to other compounds currently used for quality assurance are reasonably consistent within the whole species, and also as per genotype. Hence, the analysis of flavonoids is to certain extent an indicator for the whole phenolic spectrum. Our data suggest that a sample of underground parts containing less than 500 µg/mL of **1**(+**2**) in a derived 70% EtOH extract (corresponding to about 0.33% in the dry drug) is unlikely to be *R. rosea* or of otherwise compromised quality.

The relative variation of the main characteristic constituents according to rhizome/root proportion and provenance is also an important issue to understand the possible divergent effects reported in pharmacological or clinical studies. Summarizing our new results for flavonoids with our previous findings (rosavins and salidroside), it becomes clear that although comparing ‘Rhodiola’ with ‘Rhodiola’ may often appear seemingly the same, in fact, without the specified content of all three compound classes it is not clear which main substance group biological systems are mostly exposed to. In some cases prevail rosavins (e.g., 70% rhizome extract from a specific provenance of *R. rosea*). In other cases, flavonoids (e.g., 70% root extract from a specific provenance) or salidroside (e.g., a 30% extract from a *Rhodiola* species other than *R. rosea*) may dominate the tested drug or product and thus the pharmacological effects (Peschel et al. 2013, 2016, 2018).

### Overall range of flavonoid content

Our results show remarkably high flavonoid values in 70–90% EtOH extracts from rhizome and in particular, from roots of authentic *R. rosea*. Quantities are comparable or superior to the usual standards rosavins and salidroside, and are in the upper range of scarce previous reports. Whether similar results are obtained with different sample sets, extracts and analytical methods remains to be investigated. The first report on quantitative differences between roots (range 0.2–0.45%) and rhizomes (0.08–1.2%) of *R. rosea* came from cultivation tests in Finland, however, without specifying the flavonoids. The flavonoid content of hairy roots was found to be three times higher than that of rhizomes, and showed a decreasing trend with increasing fertilization (Galambosi 2006).

Later studies usually refer to ‘roots’ with assumed diverse mixtures of roots and rhizomes. Reported quantities for single flavonoids vary, as do species provenance, plant part, and extraction solvent (when information is available). Data are mostly obtained in the context of isolation and identification works but not from validated quantitative analysis. Overall, **1** is typically the main finding alongside **2**, rhodionin and kaempferol. Reports are available on isolations of **1** (19 mg/g) and rhodionin (17 mg/g) from ‘root’ (80% EtOH extract) (Kwon et al. 2009), of **1** (1.7–2.5 mg/g), rhodionin (0.14–0.3 mg/g), **2** (0.51–1.5 mg/g) and kaempferol (0.78–1.5 mg/g) from ‘root’ (unspecified extract) (C. Ma et al. 2013) and later of **1** (22.9 mg/g), rhodionin (13.3 mg/g), **2** (5.5 mg/g) and kaempferol (6.9 mg/g) from ‘root’ (unspecified extract) (Ma et al. 2014). Roots of *R. sachalinensis* were also investigated and **1** (7.13 mg/g) alongside rhodionin (3.98 mg/g), kaempferol (8.94 mg/g), afzelin (2.82 mg/g), multiflorin B (1.0 mg/g) and kaempferol-3,4′-di-*O*-β-d-glucopyranoside (0.4 mg/g) were detected in the dry extract (80% acetone) (Choe et al. 2012). Another study reported quantities of rhodionin in the range of 0.04–5.7 mg/g (methanol/EtOAc from ‘roots and rhizomes’) in 14 Asian *Rhodiola* species (*R. rosea* not included) without information on the content of other flavonoids (Li and Zhang 2008). Furthermore, overground parts of *R. rosea* showed **1** and **2** among 15 gossypetin, kaempferol, and quercetin glycosides (Petsalo et al. 2006). In summary, while **1** does not appear to be unique neither to the species *R. rosea*, nor to any of the plant parts, its general appearance and quantitative prevalence suggest that it could serve as a potential quality marker for *Rhodiolae roseae rhizoma et radix*. We assume that the subordinated aglycon herbacetin is a natural co-constituent of the plant rather than an artefact originating from drying, extraction or analytical processing. Based on our method no other flavonoids such as rhodionin or kaempferol were identified in our rhizome and root extracts of *R. rosea*.

## Conclusions

This is the first systematic study focusing on occurrence and amount of **1** and **2** in *R. rosea* drugs and preparations. **1** and **2** were best extracted with 70–90% EtOH, but are also detectable in more polar extracts. Variations in drying conditions did not influence the flavonoid content. We have consistently found **1** and **2** in over 100 samples of authentic *R. rosea* with major quantitative differences between rhizome and root, and influenced by plant origin despite equivalent age and growing conditions. To a lesser extent and in different ratios they were also found in 3 non-confirmed *Rhodiola* species, as well as in marketed commodities that are unlikely to contain *R. rosea* as a main source plant.

Flavonoids **1** and **2** can be detected simultaneously to phenylpropenoids and salidroside, often in concentrations higher than those. A gene pool of 20 provenances showed partially significant differences in the flavonoid (**1** and **2**) content within the overall range of 760–6300 µg/mL per extract, corresponding to approximately 0.5–4.2% (w/w) in the dry drug (exhaustive extraction). Ratios of rosavins to flavonoids (**1 **+** 2**) were 0.30–4.84 (rhizomes) and 0.10–1.54 (roots), while ratios of salidroside to flavonoids were found to be in the range of 0.06–4.91 (rhizomes) and 0.01–4.05 (roots).

Although the flavonoids **1** and **2** are also found in other *Rhodiola* species, their prevalence, their ratio to each other and to phenylpropenoids and salidrosides may be useful for quality testing. Thus, it may give an additional analytical option with an easily detectable and quantifiable marker for the specification of *R. rosea* products for medicinal use. Further research is needed to confirm occurrence and quantity of **1** and **2**, as well as other flavonoids from other data sets – potentially including other common *Rhodiola* species.
